# A Propensity Score Cohort Study on the Long-Term Safety and Efficacy of Sleeve Gastrectomy in Patients Older Than Age 60

**DOI:** 10.1155/2020/8783260

**Published:** 2020-07-31

**Authors:** Judith Molero, Romina Olbeyra, Josep Vidal, Ferran Torres, Silvia Cañizares, Alba Andreu, Ainitze Ibarzabal, Amanda Jiménez, Ana de Hollanda, Violeta Moizé, Lilliam Flores

**Affiliations:** ^1^Endocrinology and Nutrition Department, Obesity Unit, Hospital Clinic, 170, Villarroel Street, Helios Office 9, Barcelona 08036, Spain; ^2^Institutd'Investigacions Biomèdiques August Pi Sunyer IDIBAPS, 180, Corcega Street, Barcelona 08036, Spain; ^3^Centro de Investigación Biomédica en Red de Diabetes y Enfermedades Metabólicas Asociadas (CIBERDEM), Barcelona, Spain; ^4^Biostatistics Unit, Faculty of Medicine, Universitat Autònoma de Barcelona, Medical Statistics Core Facility, IDIBAPS, Hospital Clinic Barcelona, Barcelona 08036, Spain; ^5^Psychiatry and Psychology Department, Hospital Clinic, Department of Clinical Psychology and Psychobiosslogy, University of Barcelona, Barcelona 08036, Spain; ^6^Department of Surgery, ICMDM, Hospital Clinic of Barcelona, Barcelona, Spain; ^7^Gastrointestinal Surgery Department, Obesity Unit, Hospital Clínic de Barcelona, Barcelona, Spain; ^8^Centro de Investigación Biomédica en Red, Fisiopatología de la Obesidad y Nutrición (CIBEROBN), Barcelona, Spain

## Abstract

**Background:**

Bariatric surgery (BS) in older obese subjects (>60 years of age) has risen in the past decade and will continue to rise in the coming years due to ageing of the population.

**Aim:**

To evaluate the short- (12 months) and long-term (60 months) results of laparoscopic sleeve gastroscopy (LSG) in patients older than age 60.

**Methods:**

We performed a retrospective review of patients prospectively included in a database from January 2007 to December 2013. All patients >60 [older group (OG)] who had undergone LSG were included. The control group (CG) included patients aged 50 to 59 years who had undergone LSG during the same period.

**Results:**

116 (8.4 % of total surgery) and 145 patients were included in the OG and CG, respectively. BS in patients >60 years increased from 2.4% in 2003 to 14% in the last 2 years of the study. After inverse probability of treatment weighting (IPTW) analysis, all absolute standardized differences were <0.15. A 60-month follow-up was attained in 90% of patients in the OG and 74% in the CG. There were no significant differences in postoperative complications between the two groups. At 12 and 60 months after LSG, both groups achieved a similar body mass index. There was no statistical difference in the percentage of resolution of type 2 diabetes, hypertension, dyslipidemia, and SAHS between the two groups. In both groups, all the nutritional parameters evaluated remained within the normal range throughout the study.

**Conclusions:**

LSG provides acceptable outcomes and is safe in older adults indicating that age should not be a limitation to perform BS in this population.

## 1. Introduction

The incidence of obesity continues to rise especially in older adults (a population defined by the United Nations as adults aged ≥60 years) [1]. In addition, the number of older adults has considerably increased in developed countries and will likely increase further as the baby boomer generation continues to age [2]. In 2017, 25.3% of the population in Spain was older than age 60, and this prevalence is projected to increase to 41.9% by 2050 [3]. Bariatric surgery (BS) is currently the only effective treatment to achieve significant and sustained weight loss (WL) in patients with severe obesity [4].

The use of BS in older adults was initially limited due to a greater inherent risk, the lack of evidence of long-term impact of BS on weight control, remission, improvement of comorbidities, and nutritional level as well as the potential harmful effects of massive WL on muscle and bone mass [5, 6]. Nonetheless, the use of BS in subjects >60 years has risen in the last decade, representing 10% of patients undergoing BS in the United States [7]. This increase coincides with the documented safety of BS due to the incorporation of the laparoscopic approach and improvements in preoperative management in addition to the aforementioned exponential increase in obesity in this population group, which, in the coming years, will lead to a greater number of older adults requiring BS.

Previous studies have compared the safety, WL, and comorbidity resolution following laparoscopic BS in younger and older cohorts, and logically, these comparisons are affected by significant bias due to the inherent differences in overall health, with, thus, a less favorable operative risk profile in older aged subjects. In addition, studies on mid- and long-term outcomes of BS in older adults using the current surgical approaches are still scarce. Therefore, at present, the major deficiencies in the knowledge about BS in older adults do not allow firm conclusions to be drawn to help guide clinical recommendations.

Taking all of the above into account, the aim of this study was to evaluate both the short- (12 months) and long-term (60 months) evolution of anthropometric measures, comorbidities, and nutritional parameters in patients older than age 60 who had undergone LSG compared with individuals of age 50–59 who had also undergone LSG using an inverse probability of treatment weighting- (IPTW-) based analysis. This analysis reduces the impact of treatment-selection bias and the potential confounding factors inherent to an observational study, thereby facilitating adjusted comparisons between the two groups.

Our hypothesis was that LSG would provide benefits without differences between the two groups, thereby supporting extension of the upper age limit for BS.

## 2. Material and Methods

We performed a retrospective review of patients prospectively included in a database in our institution (university hospital) from January 2007 to December 2013. All patients older than age 60 (older group (OG)) who had undergone LSG as a stand-alone procedure with a follow-up of one to five years were included in the study. In order to select an appropriate older control group (CG), we included patients aged between 50 and 59 who had undergone LSG between January 2007 and December 2013 to ensure a 5-year follow-up.

All patients met the standard eligibility for BS [4]. In our institution, the criteria to perform LSG are age >60 years, a body mass index (BMI) > 50 kg/m^2^, and high surgical risk. On the other hand, laparoscopic gastric bypass (LGB) was performed in cases with a history of moderate to severe gastroesophageal reflux disease. SG was performed as described elsewhere [8]. The postoperative follow-up visits were scheduled at 4 and 12 months during the first year and yearly thereafter, and protein (minimum 60 g/day and up to 1.5 g/kg of ideal body weight/day), vitamin, and mineral supplementation were prescribed. The exclusion criteria were liver cirrhosis, chronic renal failure (creatinine ≥2 mg/dl or glomerular filtration less than 40 ml/min), follow-up less than 1 year, or no information about the primary variable of the study.

The following variables of all the patients were obtained from the database: anthropometric data, age, body mass index (BMI), presurgical comorbidities (T2D, hypertension, dyslipidemia, and sleep apnea hypopnea syndrome (SAHS)). Duration of follow-up, diagnosis, and resolution of obesity-related comorbidities were defined according to the standardized metabolic and BS outcomes of the American Society for Metabolic and Bariatric Surgery (ASMBS) recommendations [9]. The operative variables collected included the type of surgery, American Society of Anesthesiologists Score (ASA), and postoperative complications at 30 days. The complications were classified according to the Clavien-Dindo classification. Clavien-Dindo II-IIIa (adverse events requiring medication or an intervention under local anesthesia) were defined as significant, and Clavien-Dindo IIIb-V (adverse events requiring an intervention under general anesthesia) were defined as serious [10]. In addition, biochemical (hemoglobin, red blood cells, hematocrit, creatinine, uric acid, total cholesterol, c-HDL, c-LDL, triglycerides, alanine amino transferase (ALT), aspartate amino transferase (AST), gamma-glutamyl transferase (gGT), and albumin) and nutritional parameters (ferritin, transferrin, prealbumin, folic acid, calcium, magnesium, phosphorus, vitamin D3, PTH and vitamin B12) were registered. Finally, absolute body weight lost (ABWL in kg) and total body weight lost (TBWL %) were calculated at 12 and 60 months of follow-up [9]. Outcomes were assessed at the time of surgery, and at 12 months and 5 years after BS.

Loss to follow-up was assessed as misses at scheduled appointments and no attempt to contact was made.

The study was approved by the Ethics Review Board of our institution, and the need for informed consent was waived due to the retrospective nature of the investigation (HCB/2019/0641).

## 3. Statistical Analysis

A biostatistician (FT) was responsible for the statistical analyses. Categorial variables are described as frequencies and percentages and continuous variables as mean (standard deviation). We used standardized differences, defined as differences between groups divided by pooled standard deviation to assess heterogeneity between the two cohorts for baseline covariables. The IPTW approach [11] was used to create a pseudo-population in which the 2 surgery groups were balanced across baseline covariates. The stabilized weights were calculated using propensity scores [12] obtained from a logistic regression model aimed at minimizing the between arms standardized differences [13]. The covariates included in the final propensity score model were sex, BMI, excess body weight (EBW), ASA, current smoking, T2D, duration of type 2 DM, insulin treatment, hypertension, duration of arterial hypertension AHT, hypotensive treatment with more than 3 drugs, and dyslipidemia. Postbaseline variables and outcomes were available only after the definition of the final model for the IPTW. Covariate balance was assessed using the standardized differences (STD) with the initial goal of achieving values <0.10 using the IPTW to define insignificant differences in potential confounders. For baseline comparisons, the cut-off target was achieved in all variables except for sex (STD: −0.13) and SAHS (STD = −0.12), although, for some authors, these values are acceptable because the STD is less than 0.2 [14]. Baseline categorical variables were compared using the chi-square test, and continuous variables were compared using ANOVA with rank-transformed data for both raw and IPTW analyses. The primary outcome was the comparison of %TBWL between the OG and CG groups at the final time point. Secondary outcomes included the operative/30-day postoperative complication rate and remission and resolution of type 2 DM, arterial hypertension, dyslipidemia, and SAHS.

Continuous longitudinal data were analyzed using a restricted maximum likelihood- (REML-) based repeated measures approach including the fixed, categorical effects of treatment, time, and treatment by time, setting the (co) variance structure to the compound symmetry type. Longitudinal binary data were analyzed using marginal models including the same effects as for the continuous variables. The models were IPTW weighted in both longitudinal analyses.

In addition, a post hoc ancillary analysis was conducted to explore the covariate difference regardless of extreme age categories: <60 years versus ≥65 years. In this case, we used 1 : 2 matching to obtain patients with similar characteristics.

The statistical analyses were performed using SAS 9.4 (SAS Institute, Inc., Cary, NC, USA).

## 4. Results

A total of 1,375 patients underwent BS during the study period, with 128 (9.3 % of total BS) patients being older than age 60 at the time of surgery (OG). LSG was performed in 712 patients, 85 of whom were aged between 60 and 64, and 31 were more than age 65 (overall 116 patients). In addition, during the study period 196 patients of 50–59 years underwent LSG, 145 of whom met the study inclusion criteria (CG). In our institution the percentage of BS in patients older than age 60 increased from 2.4% in 2003 to 14% in the last 2 years of the study.

Based on a raw analysis, sex, current smoking, body weight (BW), EBW, and vitamin D levels were imbalanced between the OG and the CG groups. On the other hand, the two groups were comparable in the ASA score, the prevalence of hypertension, T2D, and SAHS, and there were no significant differences in the mean duration of T2D (9.38 (6.76) versus 8.25 (3.86) years, p 0.442) and the mean duration of hypertension (11.18 (6.56) versus 9.87 (6.43) years, p 0.077) between the two groups. The adjusted IPTW resulted in well-balanced OG and CG cohorts, which were similar in all the characteristics observed improving the covariate balance from the raw data cohorts. After IPTW analysis, all absolute standardized differences were <0.15. During follow-up, 11 and 12 patients in the OG and CG, respectively, underwent revisional surgery and were not included in the analysis at 60 months. A follow-up of 60 months was attained in 95/106 patients (90%) in the OG and 98/133 (74%) in the CG.  Table 1 shows the baseline characteristics of the raw and IPTW analyses in the two groups.

Table 2 shows the rates of operative and 30-day postoperative complications according to the Clavien-Dindo classification. The groups did not differ in the rate of postoperative complications. The overall rate of complications was low (9,5%), with 5 complications being considered as severe, precluding the need for statistical analysis. No surgical conversion from laparoscopic to open surgery was needed. There were no perioperative deaths.

Table 3 summarizes the postoperative WL results and improvement of comorbidities in these patients. At 12 and 60 months after LSG, both groups achieved a similar BMI. However, OG patients had a significantly lower ABWL (kg) and TBWL (%). There were no statistical differences in the percentage of resolution of T2D, hypertension, dyslipidemia, and SAHS between the two groups. Additionally, glycaemia, A1c, creatinine, liver enzymes, total cholesterol, triglycerides, c-HDL, c-LDL, and uric acid levels were similar between the 2 groups at both 12 and 60 months after LSG.

Table 4 shows the nutritional status of the two study groups. In both groups, all the parameters evaluated remained within the normal range throughout the study except for transferrin which significantly decreased at 12 months after LSG in comparison to the pre-LSG value. Indeed these values were below normal at 12 months, but they recovered to normal levels in the evaluation at 60 months, being significantly lower in the OG. In addition, red blood cells, hemoglobin, and hematocrit levels remained within the normal range and were similar in both groups. PTH significant decreased at 12 months after LSG but increased thereafter. While vitamin D levels were deficient in both groups prior to LSG, they significantly increased along the study period, reaching values close to normal ( >30 ng/ml) at 12 and 60 months.

In the additional subgroup analysis, patients ≥65 years were compared with a matched 1 : 2 group of patients <60 years. This analysis showed similar outcomes in the rate of complications, comorbidities, and even BMI (data not shown).

## 5. Discussion

The increasing prevalence of obesity in older adults illustrates the combination of two of the main burdens to healthcare systems nowadays. In this study, we report the experience of a single university center in patients older than age 60 who had undergone LSG and were followed for more than 5 years. In contrast to the previous studies, we selected a CG with preoperative medical comorbidities and surgical risk similar to those of the OG to avoid the inherent risk differences between the nonolder and older cohorts. Compared to the CG, the OG achieved less ABWL and TBWL; however, both groups obtained a similar BMI in the short and long term. The groups did not differ in the rate of postoperative complications. Indeed, the overall 30-day surgical morbidity after LSG was low, with an acceptable risk/benefit ratio in both groups. In addition, along the study period, WL was associated with improvements in all the comorbidities as well as nutritional status, which was similar in both groups.

Regarding older adult outcomes after LSG, there is a paucity of well-reported studies. The main limitations of previous studies were the differences in age in patients considered as older adults, which varied from 50 to 70 years. Moreover, most of the studies included less than 100 patients, the subjects enrolled were mainly females, there was an imbalance in comorbidities in the control groups, and nutritional status reports were poor, all of which make it more challenging to achieve a favorable balance between benefit and risk for BS in older adults [15, 16]. All the methodological limitations of previous studies have contributed to the controversy still remaining in regard to the indication of BS in older adults. In this sense, in 2013, the European Guidelines for Metabolic and BS still stated that proof of a favorable risk benefit must be demonstrated in elderly patients before surgery can be contemplated in these individuals [17], and in the NIH guidelines for BS, the upper limit for BS continues to be 60 years [18]. However, in contrast with these age restrictions, several scientific societies, such as the Italian Society for Bariatric and Metabolic Surgery (SICOB), have recently extended BS indications to elderly patients under the age of 70 [19], and the Federal Health Department in Brazil has authorized BS in individuals above 65 years without an upper age limit [20].

Similar to our findings, previous studies have also reported that older patients lose less weight. This fact might be a result of the aging process which affects their baseline physical condition, the presence of impaired muscle metabolic capacity, and a greater presence of sarcopenia that can be aggravated by the loss of lean body mass that follows after BS (described in patients undergoing BS and ranging from 10% to 25%), all of which have been negatively correlated with WL [16, 21, 22]. However, despite this lesser WL, the OG improved in all the comorbidities evaluated.

Our findings show that chronological age alone should not be an absolute contraindication for BS and confirm the efficacy and safety of LSG in this population. Moreover, our results support adjustment of the upper age limit and we even suggest that it should not be a limitation because there are no contraindications *per se* for major surgery in older adults. In older patients, the overall primary surgical goal should be to slow the age-related decline in physical function and improve their quality of life, and, therefore, potential older surgical candidates who have disabling obesity that can be ameliorated with WL should be considered. Notwithstanding, these candidates should be carefully evaluated (due to their specific needs) by a multidisciplinary team to ensure that the risk of postoperative morbidity and mortality is acceptable, and that they can gain the most benefits from this surgery.

We acknowledge that the results of observational studies are never definitive since potential confounding differences between groups cannot be completely eliminated. Although proof of the health impact of BS in older adults would require long-term randomized, controlled trials (RCTs), these are expensive, difficult to perform, and there are a number of challenges regarding the feasibility of conducting large RCT in BS in older adults and comparing the results with nonsurgical treatment. However, an alternative to a large RCT is a pooled analysis of carefully designed observational studies with the use of robust statistical methodology. Although the main limitation of the present study was its retrospective design, we followed a standardized protocol, and to ensure accurate data collection, all variables were harmonized and collected prospectively. The strengths of this study were the baseline similarities between the two study groups, including BMI and major obesity-related comorbidities after IPTW-based analysis that equated the potential confounding factors and thereby facilitated the outcome model. Indeed, the majority of variables had absolute standardized differences <0.10, and only sex and SAHS were <0.15, which allowed better assessment of the effectiveness and benefits of LSG in older adults. In addition, the sample size of both groups was adequate and with a low attrition rate.

In summary, LSG provides acceptable outcomes and is safe in selected older adults. The results of the present study indicate that age should not be a limitation to perform BS in this population.

## Figures and Tables

**Table 1:** Baseline characteristics of the raw and IPTW analyses in older and control patients.

Variables	Raw analysis				IPTW analysis			
	Control group (CG) *n*:145	Older group (OG) *n*: 116	*P*	Standardized difference	Control group (CG)	Older group (OG)	*P*	Standardized difference
Age (year)	54.8 (2.6)	63.3 (2.5)	<0.001					
Sex (female %)	89 (61.4)	97 (83.6)	<0.001	−0.514	103 (71.9)	86 (77.6)	0.294	−0.133
BMI (kg/m^2^)	47.1 (6.5)	45.3 (5.5)	0.055	−0.240	46.7 (6.0)	46.2 (5.9)	0.522	−0.081
EBW (kg)	58.6 (17.8)	50.0 (13.5)	<0.001	−0.428	56.1 (15.8)	54.5 (15.4)	0.457	−0.090
ASA	2.4 (0.5)	2.5 (0.5)	0.377	0.110	2.50 (0.5)	2.4 (0.5)	0.669	−0.053
Current smoker (%)	29 (20.0)	4 (3.4)	<0.001	0.532	18 (12.6)	8 (7.4)	0.181	−0.046
Type 2 DM (%)	63 (43.4)	62 (53.4)	0.108	0.201	69 (48.0)	51 (45.6)	0.711	−0.026
Duration DM (year)	8.2 (3.8)	9.3 (6.7)	0.442	0.094	8.9 (4.7)	8.7 (5.9)	0.609	−0.064
Insulin treatment (%)	3 (2.1)	8 (6.9)	0.053	0.234	5 (3.1)	5 (4.3)	0.624	0.061
AHT (%)	108 (74.5)	92 (79.3)	0.359	0.114	109 (76.1)	88 (79.0)	0.581	0.069
Duration AHT (year)	9.8 (6.4)	11.1(6.5)	0.077	0.221	10.7 (5.5)	10.7 (5.5)	0.872	0.020
Anti-AHT drugs: >3 (%)	28 (19.3)	19 (16.4)	0.540	−0.097	24 (16.8)	19 (17.3)	0.925	0.011
Dyslipidemia (%)	68 (46.9)	62 (53.4)	0.292	0.131	70 (478.9)	55 (49.5)	0.918	0.012
SAHS (%)	59 (40.7)	40 (34.5)	0.304	−0.128	57 (39.6)	38 (34.0)	0.358	−0.116

Data are mean (SE); BMI: body mass index; EBW: excess body weight; ASA: American Society of Anesthesiologists Score; DM: diabetes mellitus; AHT: arterial hypertension; SAHS: sleep apnea hypopnea syndrome.

**Table 2:** Operative and 30-day postoperative complications in the two groups according to the Clavien-Dindo classification.

Grade	Control group (CG)	Older group (OG)
I	4	0
II	8	5
IIIa	3	0
IIIb	1	4
IV	0	0
V	0	0
Total	11%	8%
Overall	9.5%	

Grade I: any deviation from the normal postoperative; II: normal course altered; III: complications that require intervention of various degrees; IIIa: complications that require an intervention performed under local anesthesia; IIIb: interventions that require general or epidural anesthesia; IV: life-threatening complications; V: death of a patient.

**Table 3:** Evolution of anthropometric variables and comorbidities in the two groups at 12 and 60 months after bariatric surgery.

Baseline			12 months			60 months		
Variables	CG	OG	CG	OG	*P*	CG	OG	*P*
BW (kg)	121.18 (1.31)	119.18 (1.49)	82.89 (1.31)	84.80 (1.49)	0.334	88.48 (1.42)	90.06 (1,56)	0.455
BMI (kg/m^2^)	46.70 (0.43)	46.25 (0.49)	31.92 (0.43)	32.89 (0.49)	0.141	34.07 (0.48)	35.00 (0.52)	0.190
ABWL (kg)	—	—	38.28 (0.82)	34.39 (0.94)	0.002	32.79 (0.97)	28.67 (1.03)	0.003
TBWL (%)	—	—	31.42 (0.54)	28.61 (0.62)	0.001	26.79 (0.64)	23.66 (0.68)	0.001
Waist (cm)	131.92 (0.97)	129.76 (1.12)	103.62 (0.98)	103.22 (1.13)	0.790	107.44 (1.15)	109.99 (1.33)	0.148
DM2 (%)	48.0	45.6	25.9	23.1	0.660	26.7	29.8	0.666
HbA_1c_ (%)	6.51 (0.10)	6.58 (0.11)	5.67 (0.10)	5.73 (0.11)	0.678	5.95 (0.11)	6.08 (0.12)	0.450
AHT (%)	76.1	79.0	46.1	43.0	0.613	47.7	57.1	0.671
SBP (mmHg)	137.76 (1.47)	133.85 (1.76)	124.54 (1.54)	132.57 (1.88)	0.001	136.00 (2.01)	134.55 (2.08)	0.616
DBP (mmHg)	82.44 (0.90)	80.63 (1.07)	74.57 (0.95)	76.43 (1.16)	0.217	77.07 (1.26)	76.16 (1.29)	0.614
Dyslipidemia (%)	48.9	49.5	27.4	29.8	0.931	42.1	41.8	0.724
SAHS (%)	39.6	34.0	20.3	15.5	0.401	11.3	8.7	0.557

Data are mean (SE); BW: body weight; BMI: body mass index; ABWL: absolute body weight loss; TBWL: total body weight loss; DM2: type 2 diabetes; AHT: arterial hypertension; SBP: systolic blood pressure; DBP: diastolic blood pressure; SAHS: sleep apnea hypopnea syndrome.

**Table 4:** Biochemical and nutritional parameters in the two groups at 12 and 60 months after bariatric surgery.

Baseline			12 months			60 months		
Variables	CG	OG	CG	OG	*P*	CG	OG	*P*
Albumin (g/L)	43.37 (0.23)	43.25 (0.26)	42.46 (0.23)	42.52 (0.26)	0.867	42.57 (0.28)	42.03 (0.30)	0.197
Prealbumin (g/L)	0.25(0.01)	0.24 (0.01)	0.24 (0.01)	0.23 (0.01)	0.075	0.24 (0.01)	0.23 (0.01)	0.221
Calcium (mg/dl)^*∗*^	9.56 (0.12)	10.01 (0.13)	9.67 (0.12)	9.75 (0.13)	0.651	9.51 (0.14)	9.48 (0.15)	0.870
Magnesium (mg/dl)	1.96 (0.01)	2.00 (0.02)	2.01 (0.01)	2.04 (0.01)	0.317	2.00 (0.02)	2.03 (0.02)	0.345
Phosphorous (mg/dl)	3.45 (0.04)	3.42 (0.04)	3.75 (0.04)	3.70 (0.04)	0.476	3.52 (0.05)	3.48 (0.05)	0.616
PTH (pg/ml)	86.43 (3.15)	88.74 (3.59)	62.07 (3.22)	60.34 (3.58)	0.720	83.71 (3.75)	80.24 (3.96)	0.524
25 (OH) VD (ng/mL)	15.23 (0.90)	15.31 (1.02)	27.39 (0.93)	27.47 (1.02)	0.949	25.71 (1.12)	27.47 (1.17)	0.280
Iron (mcg/dl)	72.06 (2.70)	72.34 (3.06)	90.00 (2.74)	92.94 (3.06)	0.476	92.24 (3.25)	86.28 (3.42)	0.280
Transferrin (g/L)	2.74 (0.03)	2.66 (0.04)	2.36 (0.03)	2.37 (0.04)	0.868	2.63 (0.04)	2.51 (0.04)	0.049
Vitamin B12 (pg/ml)	419.11 (30.11)	434.18 (34.23)	771.09 (30.87)	707 (34.15)	0.170	634.41 (36.35)	690.73 (38.14)	0.285
Folic acid (ng/dl)	414.76 (11.86)	392.69 (13.30)	429.36 (12.19)	450.27 (13.33)	0.247	513.79 (14.64)	516.04 (15.06)	0.914
Anemia (%)^*∗∗*^	20.85	16.80	16.51	13.66	0.484	14.04	21.15	0.599

Data are mean (ES). Cut-off for deficiency: albumin: 31–48 g/L; pre-albumin: 0.200–0.400 g/L; ^*∗*^corrected calcium: 8.5–10.5 mg/dL; magnesium: 1.8–2.6 mg/dL; phosphorous: 2.3–4.3 mg/dL; PTH: 80 pg/mL; 25 (OH) VD: <30 ng/mL; Iron: 50–170 *µ*g/dL; transferrin: 2.5–3.8 g/L; folic acid: >150 ng/mL. ^*∗∗*^Anemia: hemoglobin in males<137 g/L; hemoglobin in females<122 g/L.

## Data Availability

Dataset generated during the study can be found using this hyperlink ../ederly_Journal_of_Obesity_05052020.sav.
